# The telomeric sync model of speciation: species-wide telomere erosion triggers cycles of transposon-mediated genomic rearrangements, which underlie the saltatory appearance of nonadaptive characters

**DOI:** 10.1007/s00114-014-1152-8

**Published:** 2014-02-04

**Authors:** Reinhard Stindl

**Affiliations:** apo-med-center, Alpharm GesmbH, Plättenstrasse 7-9, 2380 Perchtoldsdorf, Austria

**Keywords:** Transgenerational telomere erosion, Chromosomal evolution, Transposable elements

## Abstract

Charles Darwin knew that the fossil record is not overwhelmingly supportive of genetic and phenotypic gradualism; therefore, he developed the core of his theory on the basis of breeding experiments. Here, I present evidence for the existence of a cell biological mechanism that strongly points to the almost forgotten European concept of saltatory evolution of nonadaptive characters, which is in perfect agreement with the gaps in the fossil record. The standard model of chromosomal evolution has always been handicapped by a paradox, namely, how speciation can occur by spontaneous chromosomal rearrangements that are known to decrease the fertility of heterozygotes in a population. However, the hallmark of almost all closely related species is a differing chromosome complement and therefore chromosomal rearrangements seem to be crucial for speciation. Telomeres, the caps of eukaryotic chromosomes, erode in somatic tissues during life, but have been thought to remain stable in the germline of a species. Recently, a large human study spanning three healthy generations clearly found a cumulative telomere effect, which is indicative of transgenerational telomere erosion in the human species. The telomeric sync model of speciation presented here is based on telomere erosion between generations, which leads to identical fusions of chromosomes and triggers a transposon-mediated genomic repatterning in the germline of many individuals of a species. The phenotypic outcome of the telomere-triggered transposon activity is the saltatory appearance of nonadaptive characters simultaneously in many individuals. Transgenerational telomere erosion is therefore the material basis of aging at the species level.

## Introduction

With the rise of modern evolutionary synthesis in the 1940s (an almost exclusively Anglo-American enterprise) mathematicians and molecular geneticists had almost completely replaced paleontologists in the scientific field of evolution. Complex mathematical models and experiments on fruit flies had been regarded superior to direct observations from the fossil record. As John M. Smith put it in a *Nature* commentary: “Since that time, the attitude of population geneticists to any paleontologist rash enough to offer a contribution to evolutionary theory has been to tell him to go away and find another fossil, and not to bother the grownups” (Smith 1984). In the 1970s and 1980s, this situation changed a little, due to the statistical work of the paleontologists Stephen Jay Gould, Jack Sepkoski and David Raup (Sepkoski 2005). The implementation of mathematics and statistics into paleontology made it much more appealing for the proponents of the gradualistic mainstream model. In particular, Jack Sepkoski’s statistical analysis showed an apparent tendency for taxa to rise, level out, and decline and these findings suggested a broader evolutionary mechanism operating at the level of families and perhaps species (Sepkoski 1984; Sepkoski 2005). As a consequence and unlike Charles Darwin had suggested, the reputation of the fossil record of providing a reliable picture of evolution became partially restored. However, the observed periodicity in the extinction patterns and the very long periods of complete stasis of a species phenotype were explained away by all sorts of complex molecular–genetic mechanisms, which would make organic evolution rather unlikely, if not impossible. It is very interesting in this context that the apparent periodicity of organic evolution has been described at least four times during the last 150 years — first by the German Ernst Haeckel, then by the American Alpheus Hyatt 50 years later, again in the 1930s by the German paleontologists Edwin Hennig, Karl Beurlen and Otto H. Schindewolf, and finally in the 1980s by the American paleontologists Stephen Jay Gould, Jack Sepkoski and David Raup. However, periodicity and saltational change did not find their way into the gradualistic mainstream theory of organic evolution, mainly due to a severe lack of convincing (genetic) explanations.

Consequently, a new genetic field emerged, evolutionary developmental biology (evo-devo), which shifted the genetic agenda to the search for mutations in developmental genes, capable of producing large and/or many phenotypic changes at once. However, it is hard to imagine how sporadic mutations in these “macromutation” genes would create anything other than lonely monsters, which would hardly ever find a compatible mate, and would again make widespread and ubiquitous organic evolution a mission impossible.

In his book *The New Evolutionary Timetable*, the American paleontologist Steven M. Stanley convincingly describes what the major problems of Darwinism and the modern evolutionary synthesis are. He states that the Pleistocene sediments at thousands of collecting sites have uncovered at least 85 % of the mammalian species living today, and therefore the fossil record can no longer be regarded as providing an incomplete picture of evolution (Stanley 1984, p. 97). Most importantly, Stanley mentions the puzzling paleontological fact that within less than 12 million years, most of the living orders of mammals developed, including such diverse animals as lions, wolves, bears, horses, rhinos, deer, pigs, antelopes, sheep, bats and whales, all having descended from a tiny animal resembling a small rodent (Stanley 1984, p. 93). He further concluded: “We can now show that fossil mammal populations assigned to a particular Cenozoic lineage typically span the better part of a million years without displaying sufficient net change to be recognized as a new species. (…) If an average chronospecies lasts nearly a million years, or even longer, and we have at our disposal only ten million years, then we have only ten or 15 chronospecies to align, end-to-end, to form a continuous lineage connecting our primitive little mammal with a bat or a whale” (Stanley 1984, p. 93). “Our only reasonable recourse is to abandon gradualism in favor of punctuated evolution, which can account for the rapid changes for which we see evidence. These changes must have been brought about by strongly divergent steps that came in rapid succession” (Stanley 1984, p. 90).

According to Thomas Kuhn, a discipline within sciences is not even receptive to a paradigm shift, unless the discipline is in a state of crisis, produced by the accumulation of critical findings that resist explanation (Kuhn 2012). Over more than a century, several natural observations have accumulated that are rather incompatible with “Darwin’s selection for the fittest reproducer” as the driving force behind speciation. Let me just highlight one of them, the seasonal migration of Eastern North American monarch butterflies, which rather screams for an explanation. Every spring these butterflies start from a small area covered by pine forests in central Mexico heading north. It takes several migrating generations of butterflies during summer to reach their northern destination, which is southeastern Canada. During fall, a new generation of monarchs migrates south to exactly the same pine forests in central Mexico their ancestors have overwintered in (Reppert et al. 2010). How can sporadic gene mutations ever lead to such a stunning multigenerational group behavior, when selection can only work on single individuals in a stable environment. A random mutation in monarchs, which sets their compass to north, is great during spring and summer, but kills the whole variant lineage of butterflies in fall. Consequently, the complete multigenerational migration pattern can only evolve in a saltatory manner at once, without any adaptation to the local environment.

## The sudden appearance of nonadaptive characters in the fossil record and the theoretical concepts of saltatory evolution

Definitely one of the most prominent figures who strongly opposed natural selection right from the beginning was the German zoologist Theodor Eimer. In 1898, he wrote that the directions of evolution cannot be selected for, because from the outset they have nothing to do with utility (nonadaptive characters). In extensive studies on butterflies, he clearly showed that new species originate in the heart of the distributional area of the ancestral forms, without separation in space, without intermediate forms and in a saltatory manner (Eimer 1898).

In the 1930s, Karl Beurlen was the first German paleontologist who presented a complete theory of saltational evolution and orthogenetic change (Rieppel 2012). He was an assistant of Edwin Hennig and was greatly influenced by his work. Beurlen summarized his concept in 1937, in his book *Die stammesgeschichtliche Grundlagen der Abstammungslehre*. Another German paleontologist, Otto H. Schindewolf, shared the same opinion regarding the empirical results and their evolutionary consequences, but rejected the vitalistic forces and the ideological overburden (Rieppel 2012). Based on his extensive studies of corals and cephalopods, he further refined the theory and presented it in his most famous book, whose publication was delayed in postwar Germany until 1950 (Schindewolf 1993). Richard Goldschmidt, a well-known German geneticist, who had to leave his country because of the Nazis, had already published, in 1940, *The Material Basis of Evolution* at the University of California at Berkeley, which in many aspects parallels Beurlen’s, Hennig’s and Schindewolf’s thoughts. He insisted that organic evolution proceeds in a saltatory manner that originates from chromosomal changes, not mutations in single genes, which never exceed the boundaries of a species. The individual outcomes he called “hopeful monsters” (Goldschmidt 1982).

Because neo-Darwinists have heavily caricatured the non-gradualistic models in the literature, I include here numerous original quotations grouped in topics, which show that saltatory evolution of nonadaptive characters seems to be a common form of “real-world evolution.” It is Schindewolf’s phylogenetic model that in my view is most advanced and closest to the world of fossilized facts. Since his work is unknown to contemporary biologists, I first present his evolutionary model followed by my elaboration on the potential biological mechanisms, which might underlie orthogenetic saltational evolution. But first, let us listen in on some statements by great observers of nature, who have no counterparts in contemporary science.

### The definition of orthogenesis by Eimer and Schindewolf

Theodor Eimer showed in extensive studies on butterflies that there is a lawfulness to the sequences of colors and patterns in every lineage, which cannot be explained by selection. In his 1898 book, Eimer refers to the term orthogenesis: “In my view development can take place in only a few directions because the constitution, the material composition of the body, necessarily determines such directions and prevents indiscriminate modification. (…) Now, in this influencing of the direction of evolution by the constitution of organisms, in this specific physiological individuality of organisms, we have the so-called inward causes of transmutation, which plainly have nothing to do with the causes assumed by Nägeli nor with his principle of perfection” (Eimer 1898, p. 22).

Schindewolf further elaborated the theory of orthogenesis: “Indeed, it is typical for orthogenesis that in many, many cases it proceeds along its controlled path in parallel lineages under the most varied, broadly fluctuating environmental conditions, causing the same sequence of character transformations to unfold” (Schindewolf 1993, p. 355). “The internal reasons for parallelism [parallel evolution] (…) reside in the matching genotypes linking the lineages in question, which allow only a limited number of possible directions” (Schindewolf 1993, p. 278). “But orthogenesis by no means comes to a halt once a biologically more favorable peak is reached. It exceeds this optimum and leads to the typolytic phase of evolution, in which unquestionably disadvantageous forms with excessive gigantism and overspecialization of individual organs develop. This evolutionary decline is difficult to understand in terms of selection” (Schindewolf 1993, p. 356).

Contrary to the predictions of genetic gradualism, the amount of morphological novelty has been recently found to decline steadily over time in a lineage, completely uncoupled from the oscillating levels of species diversity (Ruta et al. 2013). Yet, Schindewolf had already written in 1950: “(…) the set of rudiments in the first representatives of each lineage largely determines later evolution, and that subsequent differentiational steps entail a progressive narrowing of evolutionary creative potential” (Schindewolf 1993, p. 273). Schindewolf adapted the law of progressively reduced variation, which was initially proposed by the Italian zoologist Daniele Rosa, another proponent of orthogenesis. Rosa hypothesized that evolution is written within living beings in the same way that future development is written within the first cell of an organism (Luzzatto et al. 2000). Rosa seemed to have some impact on the writings of Hennig (Luzzatto et al. 2000), who in turn influenced Beurlen and Schindewolf.

“Being suspicious of mysticism and vitalism, Schindewolf argued strongly against a teleological, finalistic interpretation of orthogenesis. In his view, orthogenesis is not directed toward a goal but is rather constrained from its particular starting point” (Afterword by Wolf-Ernst Reif in Schindewolf 1993, p. 447).

### The limited powers of selection and adaptation as seen by Eimer and Schindewolf

Eimer refers to nonadaptive characters: “The directions of evolution cannot possibly be selected, for the reason that from the outset they have nothing whatever to do with utility” (Eimer 1898, p. 24). “I must, in fact, reiterate again and again that natural selection (…) can only work with existing material, and it cannot even use that until it has attained a certain perfection, until it is already useful” (Eimer 1898, p. 21). He further concluded from the puzzling appearance of the oak leaf butterfly: “Kallima’s resemblance to a leaf is determined by a thousand and one details. Not one accident but a thousand accidents together would have been requisite, and would have had to present themselves suddenly, in order to produce this resemblance by the selectional agency of Darwinism. The resemblance to a leaf could not have gradually arisen by selectional means; it must have originated suddenly and in approximate perfection in order to have given selection any hold for its operations” (Eimer 1898, p. 54).

In accordance with Eimer, Schindewolf writes: “Adaptation controlled by selection therefore forms the conclusion of individual evolutionary cycles but never their beginning” (Schindewolf 1993, p. 349). “Moreover, as has already been mentioned, only the fact that adaptive specialization takes place only within the scope of individual, mutually exclusive types and does not exceed the boundaries of the type explains why, today, the more primitive types still exist side by side with the advanced ones, for example, why protozoans still live alongside mammals, why algae exist with flowering plants. Otherwise, if there were no such typal restrictions on selection, the more primitive lineages would long since have been replaced by the more advanced ones or been absorbed by them” (Schindewolf 1993, p. 349).

### Two different entities in the fossil record — type formation of higher families, orders and classes versus species formation

Schindewolf states: “It has been observed that during evolution there is a clear separation between basic structural characters, which arose discontinuously, introduced the new type, and became the common property of all of the members of that type, and the subordinate specific, characters, which affect only certain individual groups and exhibit gradual change in small individual steps” (Schindewolf 1993, p. 164). “Thus, the indisputable fact exists that in ontogeny, the particular builds developmentally, physiologically and morphologically, upon the general. Correspondingly, it must be the same in evolution, that the organic structure of the comprehensive types is formed before that of the types subordinate to them. This is also shown to be true by the following consideration: Every phylogenetic–taxonomic category of higher rank shows a broader geographic range, and its representatives occupy a correspondingly greater diversity of habitats than is the case with the lower categories contained within it. (…) it would be impossible, however, given the extreme variety of environmental factors, for selection to result in the formation of common structural characters, for the genera and families to merge later, secondarily, to form the typal unit of the order, and the orders to merge to form the structural design of the class. Since they all have the same basic type, it must have been laid down before the splitting into subtypes. From all this, only one logical conclusion can be drawn, and it supports our concept: the higher categories do not form additively and gradually; they are not assembled from lower categories” (Schindewolf 1993, pp. 228–229).

“Moreover, the conspicuously low number of major structural types in the plant and animal kingdoms speaks for the fact that their production must be attributed to very rare, radical events in the overall course of evolution. If the major types had arisen simply through the accumulation of numerous small remodelings of characters, which went on endlessly and randomly in all directions, they would not be so sharply limited in number and kind.” (Schindewolf 1993, p. 342) “Consequently, evolution does not proceed from the particular to the general, but in reverse, from the general to the particular, until finally a new, far-reaching transformational step casts off the specialized forms and creates another broad base for a new point of departure” (Schindewolf 1993, p. 342).

“Only the formation of types of lesser rank (…) involving insignificant quantitative differences, corresponds to the usual notion of smooth, gradual transformation, which until now has been commonly thought applicable to evolutionary processes as a whole. (…) Our experience, gained from the observation of fossil material, directly contradicts this interpretation. We have found that the organizing structure of a family or an order did not arise as the result of continuous modification in a long chain of species, but rather by means of a sudden, discontinuous direct refashioning of the type complex from family to family, from order to order, from class to class. The characters that account for the distinctions among species are completely different from those that distinguish one type from another. (…) which can only be accomplished through a sudden leap, during a very early juvenile stage. (…) and thus, with one stroke, a new, complex typal organization arises, one completely different from and directly in opposition to the ancestral type” (Schindewolf 1993, pp. 214–215). “Once the basic forms of the individual structural designs had been established, however, a continuous, slow evolution set in, which can be clearly followed thanks to the presence of closed series of gradually changing forms” (Schindewolf 1993, p. 124).

“The theory of typostrophism presented here, of an abrupt, discontinuous, complete transformation of type at various levels, is founded on paleontological evidence. Developed essentially by Karl Beurlen and the author [Otto H. Schindewolf], (…)” (Schindewolf 1993, p. 224).

### Schindewolf’s typostrophic theory

**Fig. 1:**
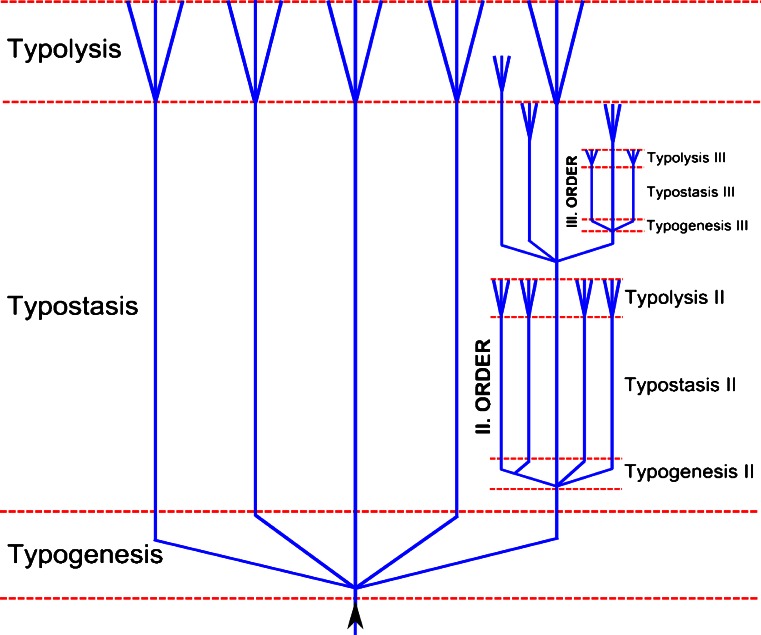
Schindewolf’s typostrophic theory as published in 1950 (Schindewolf 1993, p. 202). During the short typogenetic phase, the type breaks up into subtypes with new body plans. The lengthy typostatic phase is characterized by phenotypes, which remain rather stable with only smooth and gradual transformations. In the brief, final typolytic phase, these subtypes produce all kinds of degenerative offshoots. During the typostatic phase, evolutionary cycles of lesser rank branch off and during their typostatic phase other branches appear that go through typogenesis, typostasis and typolysis, and so on. (Figure adapted from Schindewolf 1993)

According to Otto H. Schindewolf, evolutionary development is episodic, it proceeds in phases or in quantum leaps and it exhibits a pronounced periodicity. Based on his typostrophic theory, three phases of differing evolutionary rates can be distinguished: typogenesis, typostasis and typolysis (Fig. 1). During the first brief phase of typogenesis, there is an abrupt development of forms; all different kinds of structural organizations are formed explosively in large transformational steps. Typostasis, the second phase, is characterized by progressive elaboration, diversification and differentiation within the framework of the basic form but does not alter the basic structural design itself and does not create anything essentially new. Evolution is slow and this phase lasts much longer than the other two phases. The third phase, called typolysis, displays multiple indications of decline and degeneration, like overspecialization, gigantism and abnormal forms. It is also characterized by some kind of pseudovariability, which is thought to be destructive.

According to Schindewolf, after the third phase, the more specialized representatives of the older type become extinct and the transformation to the new type takes place in a single surviving lineage. Based on his studies on the abundant fossil record of corals and cephalopods, Schindewolf concluded: “The three phases of evolutionary cycles described by several authors have their counterpart in the developmental stages of the individual cycle — in the youth, maturity, and old age of every single living being” (Schindewolf 1993, p. 194). Haeckel was the first to recognize these three phases and called them Epacme, Acme and Paracme, 150 years ago. Karl Beurlen had already introduced, in 1932, the same classification as Schindewolf. Unfortunately, his work was heavily influenced by Nazi propaganda and his favoring of vitalistic forces was rejected by his scientific colleagues.

### Goldschmidt’s material basis of saltatory evolution

In his book, *The Material Basis of Evolution*, the German geneticist Richard Goldschmidt states: “The change from species to species is not a change involving more and more additional atomistic changes, but a complete change of the primary pattern or reaction system into a new one, which afterwards may again produce intraspecific variation by micromutation” (Goldschmidt 1982, p. 206). “Microevolution does not lead beyond the confines of the species (…) Species and the higher categories originate in single macroevolutionary steps as completely new genetic systems. The genetical process, which is involved, consists of a repatterning of the chromosomes, which results in a new genetic system. (…) such genetic changes affect early embryonic processes and automatically entail major deviations in the entire organization” (Goldschmidt 1982, pp. 396–397). “The idea of the reaction system (…) means that the germ plasm as a whole, i.e., predominantly the chromosome complex, controls the general features of development which lead to a definite type, the species in question. (…) It considers only a single unit action of the whole germ plasm, with more or less independent action of the individual chromosomes” (Goldschmidt 1982, p. 218).

## Chromosomal speciation

The chromosomal theory of evolution has always been handicapped by a paradox, namely, how speciation can occur by means of gross chromosomal rearrangements that are known to decrease the fertility of a spontaneously occurring heterozygotic carrier (Walsh 1982; De Grouchy 1987). For any chromosomal rearrangement to replace the original throughout a population, the new rearrangement must increase in frequency, despite its deleterious effect on fertility, and produce new homozygotic carriers that are better adapted than the original homozygotes. Yet, the frequency of such replacement episodes seems to be extremely low, otherwise groups of individuals within a population would frequently be found to differ in chromosome complements, which is clearly not the case. However, differing chromosome complements, with identical sets of two homologous autosomal chromosomes each, are the hallmarks of diploid species and any thoughtful speciation theory has to provide a mechanical explanation, independent of considering mutations or structural chromosomal rearrangements as being the cause of speciation. Chromosomes have been found to differ in almost all cases between species (White 1968, 1978, p. 324), despite the reproductive disadvantage of heterozygotes; it is, therefore, highly unlikely that chromosomal changes just occurred by chance and are not important for speciation.

As King writes: “For the moment it should be stated that there is no longer any room for debate as to whether profoundly negatively heterotic chromosomal rearrangements can reach fixation in derived populations, the fact of their fixation is undeniable. It is up to the opponents of chromosomal speciation to explain how these rearrangements have reached fixation. That is, our concepts of population genetics must be modified to account for the existing phenomena” (King 1993, p. 122).

Most mammals, birds, reptiles and amphibians are distinguished by such chromosomal rearrangements as centric fusion and fission, pericentric inversion, tandem fusion, reciprocal translocation and the addition of heterochromatin (King 1990, pp. 147–148). Centric fusions of acrocentric chromosomes and centric fissions of metacentric chromosomes can explain the diverse range of chromosome numbers observed in many animal taxa (Perry et al. 2004). Mammalian karyotype evolution is characterized by the reshuffling of large conserved chromosomal segments. Many breakpoints are associated with the formation of acrocentric and metacentric chromosomes during evolution (Ferguson-Smith and Trifonov 2007). Extensive studies in Caudata and Anura uncovered a general trend toward symmetrical karyotypes, in which the chromosome number decreases and all the acrocentrics form metacentric chromosomes in higher families (Morescalchi 1975; King 1990, p. 145; Vignali and Nardi 1996). As has been argued by King, once metacentricity has been reached in a lineage of species that evolves by fusion of acrocentrics, chromosomal evolution may have reached a dead end (King 1990, p. 148). Because of the common occurrence of centric fusions of acrocentrics (=Robertsonian fusion or translocation) (Imai et al. 1988), but the rareness of spontaneous centric fission events (Perry et al. 2004), it was concluded that fissions of most if not all metacentric chromosomes happen simultaneously at the end of an acrocentric fusion sequence in a lineage (karyotypic fission theory) (Todd 1970; Kolnicki 2000). In this centric fusion–fission cycle, pericentric inversions are thought to be responsible for the more complex chromosomal rearrangements (Todd 1970; Imai et al. 1988). The domestic dog has an all-acrocentric karyotype (except for the sex chromosomes), whereas another canid the red fox has an all-metacentric karyotype. Eight of the fox metacentric chromosomes are interpreted as fusions of two dog acrocentrics, seven by fusions of three dog chromosomes and one by more complex rearrangements (Ferguson-Smith and Trifonov 2007). In my view, the domestic dog is a perfect example of a karyotypic fission event.

In the following, I list a collection of speciation events accompanied by fusions, fissions and pericentric inversions of chromosomes, to show how ubiquitous this phenomenon is. Robertsonian fusions have been reported to be the prevailing mechanism for the autosomal evolution of bovids (Iannuzzi et al. 2009). Horses are separated from zebras by Robertsonian fusions, tandem fusions and inversions. The domestic horse differs from the Przewalski horse by one Robertsonian fusion (Yang et al. 2003). Similarly, independent fusions of ancestral acrocentric chromosomes have led to the karyotypes of the giant panda and the spectacled bear (Tian et al. 2004). The chromosome number between two Galagos species, *Otolemur crassicaudatus* and *Galago mohohli*, differs dramatically. Stanyon et al. (2002) convincingly described ten Robertsonian fusions and two fissions underlying their genomic divergence. In canids, the principal mechanism of karyotype evolution has been chromosome fusion (Graphodatsky et al. 2000). Robertsonian fusions and pericentric inversions are involved in karyotype evolution in the family Didelphidae, characterized by the richest number of taxa of all marsupials (Svartman and Vianna-Morgante 1998). The reduction of chromosome numbers in giraffes (from 58 to 44) could be primarily attributed to extensive Robertsonian fusions of ancestral acrocentric chromosomes (Huang et al. 2008). In hares and rabbits (order Lagomorpha) chromosomal fusions and fissions shaped genome evolution (Robinson et al. 2002). Likewise, the fission–fusion process of karyotype evolution is dominant in the kangaroo group (O'Neill et al. 1999). Chromosomal evolution in bats, in the family *Vespertilionidae*, proceeds by Robertsonian fusions — less frequently centric fissions or inversions occur (Volleth et al. 2001). Despite the rather stable karyotypes of most birds, Robertsonian fusions and fissions seem to be the most common rearrangements (De Oliveira et al. 2008; Ellegren 2010). In Atlantic Anguilliformes fishes, although only a few representatives have been karyotyped, the data confirm pericentric inversions and/or Robertsonian rearrangements being the source of karyotype diversification (Vasconcelos and Molina 2009). In the plant family Zamiaceae, centric fusions and fissions dominate karyotype evolution (Olson and Gorelick 2011). The list could go on endlessly, but I will stop here and just close by mentioning the frequent reports of remnants of telomeric sequences at sites of chromosomal fusions (Ijdo et al. 1991; Slijepcevic 1998; Svartman and Vianna-Morgante 1998).

**Fig. 2:**
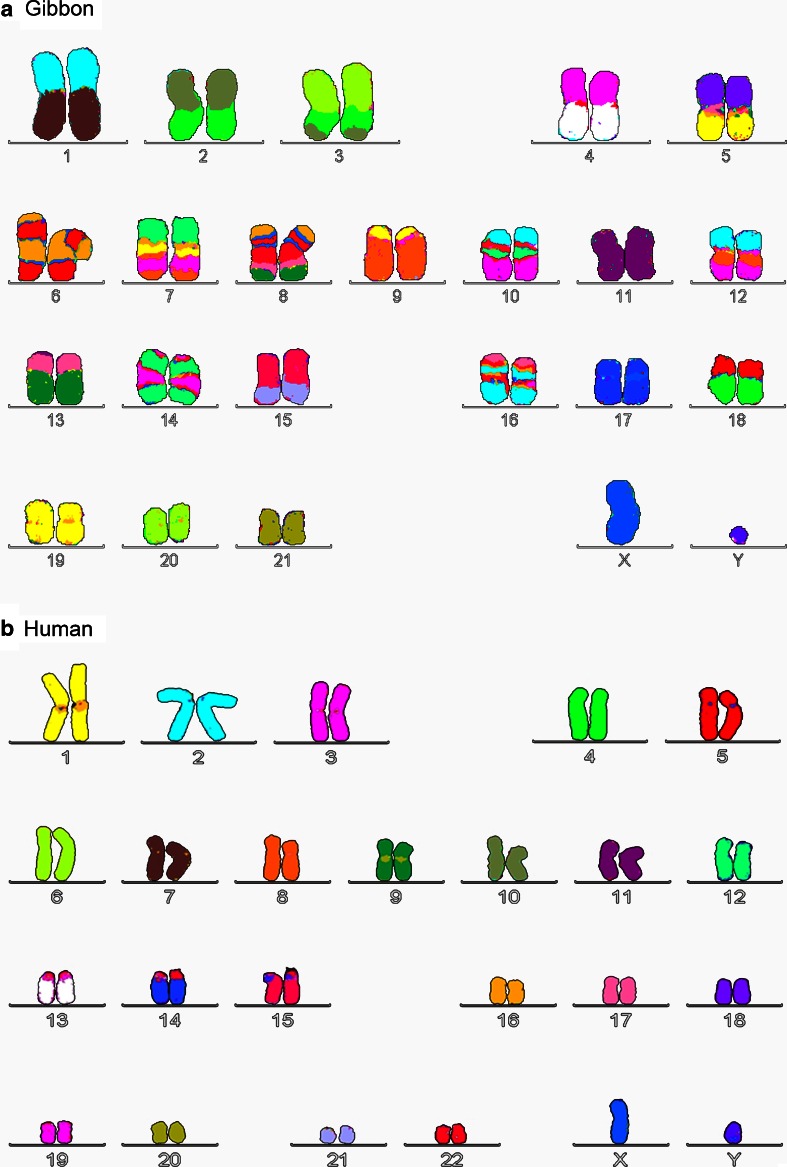
How many bottlenecks are required to generate this kind of karyotypical mess between closely related species? **a** Gibbon (*Hylobates lar*) karyotype in comparison to **b** human karyotype based on corresponding color code. Every sporadic chromosomal aberration reduces fertility of heterozygotes due to loss of genetically unbalanced offspring. So how could fixation of these massively reorganized karyotypes ever occur based on standard models (Jauch et al. 1992)? Fluorescence in situ hybridization was performed on metaphase chromosomes of a male gibbon and a male human with human multicolor FISH probes from Metasystems according to the suppliers protocol except for a slightly reduced temperature at the post-hybridization wash for the gibbon. (Unpublished result of a hybridization experiment performed by the author at the University of California at Berkeley in January 2002. The gibbon cell line was kindly provided by Johannes Wienberg at the Institute of Human Genetics at the Ludwig-Maximilians University in Munich and by Christa Lese Martin and Lorraine May at the Department of Human Genetics at the University of Chicago)

Max King writes: “There is now little doubt that chromosomal evolution in terms of the site of chromosome breakage and the type of chromosome rearrangement established is a nonrandom process” (King 1993, p. 123). De Grouchy emphasized that different branches of the phylogenetic tree evolved through specific types of rearrangements, which are pericentric inversions in the great apes, fissions in the cercopithecine monkeys, and Robertsonian fusions in the lemurs. He states: “This is a very remarkable and fundamental observation. It raises the possibility of evolution occurring in an organized fashion rather than by tinkering, but the underlying mechanisms are not obvious” (De Grouchy 1987). Similarly, White’s rule of karyotypic orthoselection implies that in many evolutionary lineages chromosome after chromosome undergo the same type of structural change, so that they all attain a similar morphology. Thus, chromosomal fusions dominate in species with many acrocentric chromosomes, whereas pericentric inversions (and fissions) are common in karyotypes with many metacentric chromosomes (White 1973, pp. 450–452). For example, the chimpanzee and human genomes (both are mostly metacentric) are distinguished by nine large pericentric inversions and one fusion of two acrocentrics that give rise to human chromosome 2 (Ijdo et al. 1991; Kehrer-Sawatzki and Cooper 2007). Yet, in none of the inversion breakpoints has any interrupted or newly created gene been found and therefore the reasons underlying the homozygous fixation of these inversions remain an enigma to molecular genetics (Kehrer-Sawatzki and Cooper 2007, 2008). Consequently, a new model of chromosomal speciation was put forward based on reduced recombination in rearranged chromosomes (Rieseberg and Livingstone 2003). The story goes that reduced recombination will lead to an accumulation of selected mutations, which facilitate hybrid incompatibility, and enables the completion of speciation. This concept seems to be flawed, since from the outset the negative effect of chromosomal aberrations on fitness prevent any significant spread of carriers in a population, and therefore there is no material for mutation and selection to work on. Clearly, any complete evolutionary theory has to explain species-specific chromosomal aberrations, because these kind of genomic changes have happened numerous times. There has to be a reason for the widespread phenomenon of gross chromosomal differences between species, if despite their being deleterious to the offspring of spontaneous heterozygotes they still make it to homozygosity in millions of new species. The currently prevailing concepts of population genetics become even more unsatisfactory, if one considers the enormous number of population bottlenecks required to explain the massive chromosomal changes in animals like the gibbons. A minimum of 33 translocations seem to have occurred in *Hylobates syndactylus* and 28 in *Hylobates lar* since their separation from humans (Koehler et al. 1995). Based on the bottleneck concept of molecular–genetic speciation models, the population sizes of gibbons must have been oscillating like a yo-yo (Fig. 2).

## Transgenerational telomere erosion triggers chromosome fusions and transposon-mediated genomic rearrangements

In 2004, I presented an alternative evolutionary model based on transgenerational telomere erosion and recurrent cycles of chromosomal instability, which result in either chromosomal fusions (and new species) or telomere stabilization or species extinction (Stindl 2004a). The so-called species clock hypothesis is based on the idea that telomere erosion synchronizes speciation events in many individuals, similar to such biological phenomena as the mass flowering events of bamboo, which occur (up to) every 120 years (Janzen 1976). All plants of the same stock flower just once at the same time, regardless of differences in geographic locations or climatic conditions (Seifriz 1950). Clearly, long-term biological clocks exist in nature, despite the lack of any good Darwinian explanation. This is also in line with the concept of the Russian biologist Leo S. Berg, who proposed an orthogenetic theory named nomogenesis, where he states that the mutational production of new forms proceeds periodically and epidemically in many individuals of a species (Berg 1969).

**Fig. 3:**
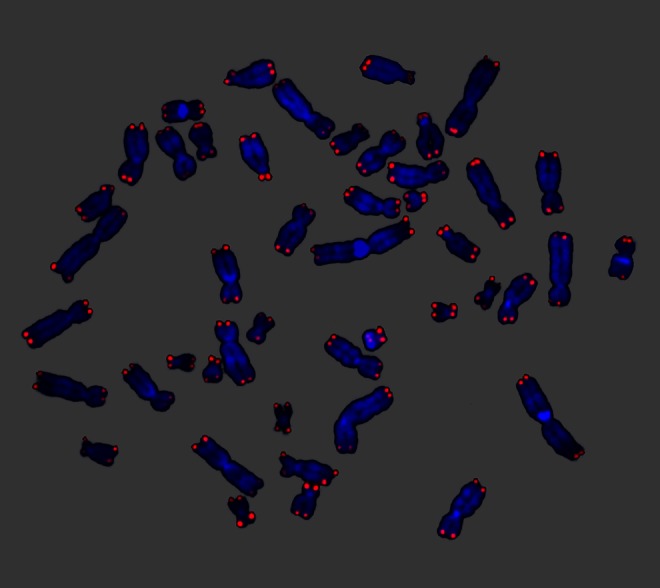
Telomeres are the protective caps at the ends of eukaryotic chromosomes. Telomeres (*red*) on human chromosomes (*blue*). PNA-FISH was performed by the author, according to standard protocols (Daco) at the Medical University of Vienna in 2006

Whereas the topic of my previous paper was mostly on telomere erosion and species extinction, this time I want to focus on the intrinsic factors of speciation and saltatory phenotypic change. Although I recommend two of my original papers (Stindl 2004a, 2011) for further study, here I briefly recapitulate the main points. Telomeres are the protective caps of eukaryotic chromosome ends (Fig. 3) and it has been shown that critically short telomeres result in chromosomal instability (Londono-Vallejo 2004). Based on the assumption that telomeres shorten between generations (Stindl 2004a) and the fact that certain chromosome arms tend to have the shortest telomeres in a species (Graakjaer et al. 2006), it was proposed that the same chromosomal fusion product appears independently in several individuals’ germline, leading to homozygous offspring without the underdominance problem of heterozygotic carriers (Stindl 2004a).

**Fig. 4:**
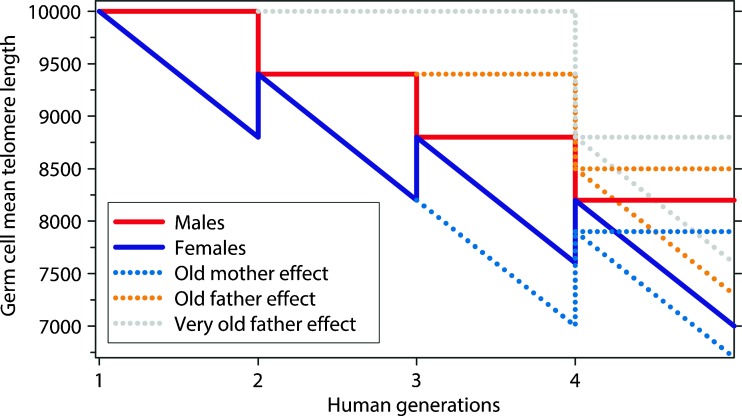
The theoretical concept of transgenerational telomere erosion in the female germline with a carry-over effect for both sexes is in perfect agreement with published data of large multigenerational studies on healthy individuals. Contrary to the mainstream view of a significant telomere length increase in the testes of very old men, I suggest that the old-father-long-telomered-offspring effect (Eisenberg et al. 2012) strongly point to ever-shortening telomeres in the female germline. According to my interpretation, telomerase basically stabilizes telomeres in testes and, therefore, the offspring of old fathers bypass the telomere loss of one female generation (figure reprinted from Stindl 2011). Very old fathers tend to have younger wives (because of social and biological reasons), whereas older mothers usually have husbands of similar advanced age, possibly resulting in a reduced loss of their offspring’s telomere length. Therefore, the negative age effect of older mothers on their offspring’s telomeres was only found in one large study, where the authors carefully adjusted for paternal age at conception (Prescott et al. 2012)

Telomeric DNA shortens in somatic tissues during a human lifetime and replicative telomere erosion is thought to be causally involved in aging (Aubert and Lansdorp 2008). So, it came as a surprise when several groups reported a significant positive correlation between paternal age and chromosome telomere length in offspring (Unryn et al. 2005; De Meyer et al. 2007; Kimura et al. 2008). In other words, what they have found is that very old fathers resulted in long-telomered offspring. Since long telomeres in normal somatic tissues are a marker for biological health, it seems to be advantageous to have a very old father (Aviv and Susser 2013). This positive age effect was really surprising, and it was concluded by the authors that telomeres significantly lengthen in the testes of very old men (De Meyer et al. 2007), although this explanation contradicts the biological fact that nothing gets better with advanced age. Alternatively, based on the correlation between maternal age and the incidence of aneuploid pregnancies (e.g., Down syndrome) (Hassold and Chiu 1985) and the fact that most oocytes of older women are chromosomally abnormal (Hassold and Hunt 2001; Pellestor et al. 2003), I hypothesized that telomere erosion predominantly operates in the female germline, leading to a carry-over effect for both sexes into the next generation (Fig. 4) (Stindl 2011). Keefe’s telomere theory of reproductive senescence elaborates on telomere erosion in the female germline, but he does not speculate on any cumulative effect between generations (Keefe et al. 2006). Yet, decisive support for my claim of transgenerational telomere erosion comes from a large human study spanning three healthy generations, published in *PNAS* (Eisenberg et al. 2012). Eisenberg and colleagues found that the positive telomere effect of older fathers is cumulative between generations. In the paternal line, older grandfathers contributed equally to longer telomeres in grandchildren as older fathers did. Maternal and grandmaternal ages were not significantly associated with the child’s or grandchild’s telomere length. However, it was found that the grandfather’s positive effect on grandchildren’s telomere length diminished in the maternal line. This is most significant, since the lengthening of telomeres in the testes of old men, the currently accepted mainstream model (De Meyer et al. 2007), is incompatible with the observed loss of the positive grandfather effect in the maternal line. In my view, the only conclusive explanation for Eisenberg's puzzling data is that telomeres not only do not lengthen in the testes of old men, but conversely shorten in the female germline resulting in ever-shortening telomeres in the human species. According to this idea, old men result in long telomered offspring because the enzyme telomerase basically stabilizes telomeres in testes and therefore the offspring of old fathers bypass the telomere loss of one female generation (Fig. 4) (Stindl 2011). Very old fathers tend to have younger wives (because of social and biological reasons), whereas older mothers usually have husbands of similar advanced age, possibly resulting in a reduced loss of their offspring’s telomere length. This seems to be the reason why the negative age effect of older mothers on their offspring’s telomere length was only found in one large study of healthy individuals, where the authors carefully adjusted for paternal age at conception (Prescott et al. 2012).

However, if telomere erosion within and between human generations is largely restricted to the female germline, the Y chromosome should not be affected (Stindl 2011). Yet, telomere lengths of the Y chromosome are comparable to those of other chromosomes of a particular species, and therefore a type of molecular trimming during early embryonic development would be required to explain this observation. In human male embryos, such an in vivo process has been described exclusively for sex chromosomes (Perner et al. 2003), where telomere lengths of the long arms of the X and Y chromosomes appear to become homogenized, possibly due to interchromosomal recombination of subtelomeres. An alternative scenario for the average telomere length of the Y chromosome is that intergenerational telomere erosion proceeds slowly in male and female germlines (Stindl 2004a), and accelerated erosion in one of the two sexes is only a late-stage characteristic of an “aged” species.

To explain pericentric inversions and other structural chromosomal aberrations (besides fusions of acrocentrics) and the saltatory phenotypic change that occurred during evolution, I realized that it is time for an upgrade of my species clock hypothesis (Stindl 2004a). In the following section, I will present an advanced version, but first comes a survey of the supportive data from the literature.

Whereas it is widely known that short telomeres can lead to fusion of acrocentric chromosomes (Blasco et al. 1997; Slijepcevic 1998), the mechanical explanation of how a (pericentric) inversion in the middle of a metacentric chromosome can be triggered by short telomeres on chromosome ends seems to be quite a challenge. Rescue comes from the Nobel laureate Barbara McClintock. Based on her experiments in the 1940s she realized that a single ruptured end of a maize chromosome (without a functional telomere) was responsible for the activation of several transposable elements, which restructured the genome on various levels — from small changes involving a few nucleotides, to gross chromosomal aberrations like deletions, duplications, inversions and other more complex reorganizations (McClintock 1984). In a pivotal *PNAS* article, Scholes and colleagues (Scholes et al. 2003) describe the direct activation of *Ty1* retrotransposons by critically short telomeres in *Saccharomyces cerevisiae* as part of the normal cellular response to telomere dysfunction. Similarly, transposable elements have been found associated with chromosomal rearrangements in insects and vertebrates (Lim and Simmons 1994; Bohne et al. 2008). In *Drosophila melanogaster*, 60 inversion breakpoints have been studied, and all but one of these contained the same DNA transposon, namely, *hobo* (Lim and Simmons 1994). According to another *Drosophila* study, approximately 85 % of the breakpoints of chromosomal rearrangements occur at the positions of *P* elements and some of the chromosomal inversions were capable of reverting to the original sequence (Engels and Preston 1984). In humans, a species-specific inversion on the Y chromosome was studied and was found to be flanked by breakpoints containing *LINE-1* retrotransposons (Schwartz et al. 1998). *LINE-1* (*L1*) is the most abundant self-replicating class of human transposons (Han and Boeke 2005), whereas short interspersed nuclear elements (SINEs), mostly *Alu* and *Alu*-like elements, depend on other transposons, for example *L1*, for their mobility (Prak and Kazazian 2000). In comparisons of the structural chromosomal changes that distinguish human and chimpanzee, segmental duplications (SDs) at the flanking regions of 70–80 % of inversions were found, and *Alu* elements were enriched at SD junctions (Bailey et al. 2003). Similarly, the breakpoint regions of chromosomal rearrangements distinguishing northern white-cheeked gibbon from human are enriched in SDs and repeats, with *Alu* elements being the most abundant (Carbone et al. 2009). In the human genome, over one million *Alu* sequences have been identified (Bohne et al. 2008). Besides *Alu*s, the *L1* retrotransposons are very common in mammalian genomes, with, e.g., 660,000 copies in the mouse, 520,000 in human and over 1.1 million copies in the short-tailed opossum (Bohne et al. 2008). The difference in activity of these *L1* transposons is thought to correlate with the number of intact and retrotransposition-competent elements, estimated at around 3000 in the mouse compared to less than 150 in human (Bohne et al. 2008). Three retrotransposon families remain mobile in the human genome: *L1*, *Alu* and *SVA* (Upton et al. 2011). Many of the large pericentric inversions distinguishing human and chimpanzee karyotypes have breakpoints in transposable elements or transposable element-rich regions (Bohne et al. 2008). Goidts and colleagues present evidence for a nonrandom model of chromosomal evolution in showing that a homologous pericentric inversion in chimpanzee and gorilla has slightly different breakpoints, consistent with independent origins in both species (Goidts et al. 2005). Not surprisingly, the breakpoints were again associated with *LINE* and *Alu* elements (Goidts et al. 2005). Several authors suggested that chromosomal speciation through transposon-mediated pericentric inversions might have played a role in the divergence between human and chimpanzee (Bohne et al. 2008; Lee et al. 2008). Regarding the submicroscopic genomic rearrangements, *L1* and *Alu* elements have been described as being responsible for 44 % of the 252 inversion loci between human and chimpanzee (Lee et al. 2008). Transposon diversity and abundance is highly variable from one species to another, and reflects their specific genome-transposon history (Hua-Van et al. 2011). Each eukaryote has a specific complement of recently active transposable elements that are key genetic features distinguishing related species (Burns and Boeke 2012).

Ever since the discovery that more than one third of the DNA of higher organisms consists of hundreds of thousands of repeated sequences (Britten and Kohne 1968) and some indications for saltatory replications and transpositions of new variants during speciation, their causal role in phenotypic change has been discussed (Britten and Kohne 1968). In 1982, Gillespie, Donehower and Strayer proposed the term “genome resetting” for the periodical reorganization of the genome by newly amplified repeated DNA, which establishes new genetic programs in development and defines the phenotype of the new species (Gillespie et al. 1982). Later, James A. Shapiro called it a fundamental aspect of speciation and claimed that this process can occur without major changes in the protein coding sequences. According to him, genomes are built up Lego-like out of codons specifying protein domains, and evolutionary genetic change is largely a matter of nonrandom codon reorganization by natural genetic engineering mechanisms like retrotransposition (Shapiro 1992, 2010). In 2011, Oliver and Green proposed a very similar model and called it the “TE-Thrust hypothesis” (Oliver and Greene 2011). All three concepts, genome resetting, natural genetic engineering and the TE-Thrust hypothesis automatically impose a punctuated tempo on the process of evolutionary change (Gillespie et al. 1982; Shapiro 1992, 2010; Oliver and Greene 2011), but all of them lack an effective trigger for the simultaneous change in several individuals of a species. Without a species-wide sync mechanism, the individual with its grossly reorganized genome will be very lonely and hence severely impaired in spreading its new type effectively in a population.

Fortunately, such a trigger mechanism is part of my telomeric sync model of speciation, which is based on transgenerational telomere erosion (Stindl 2004a) that, in combination with the observed species-specific telomere profile (Graakjaer et al. 2006), causes identical chromosomal fusion products in many individuals, simultaneously. In addition to telomere-driven fusion of acrocentric chromosomes, I propose that critically short telomeres on the ends of metacentric chromosomes trigger transposon-mediated chromosomal rearrangements, preferentially large pericentric inversions. All these types of gross chromosomal rearrangements are known to cause reproductive barriers at various levels (Jacobs et al. 1975; De Braekeleer and Dao 1990) and are therefore regarded as the causal agents of a species split. To explain instant and significant phenotypic change during speciation, I suggest eroded telomeres being the trigger of a submicroscopic transposon-mediated repatterning of the genome, which is thought to restructure the body plan of a new species. This will be further elaborated in the next chapter.

**Fig. 5:**
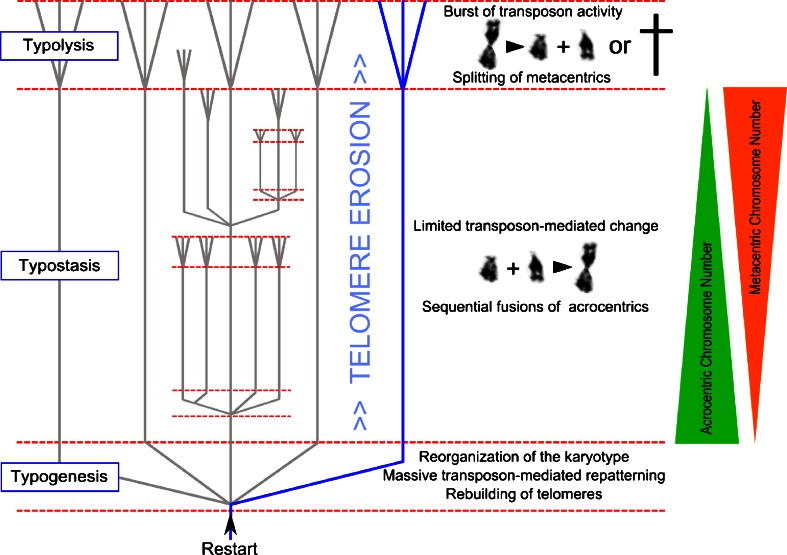
The telomeric sync model of speciation and Schindewolf’s three phases of typostrophic theory exemplified by the widespread centric fusion–fission cycle of acrocentric chromosomes. **a** Typogenesis: after the splitting of metacentric chromosomes in the typolytic phase, telomeres are rebuilt and lengthened at the beginning of the typogenetic phase. The high frequency of eroded telomeres at the transition phase between typolysis and typogenesis triggers a massive transposon-mediated repatterning of the genome that explosively creates the new body plans of new species during typogenesis. **b** Typostasis: transgenerational telomere erosion leads to the sequential fusions of acrocentric chromosomes with only limited transposon-mediated genomic repatterning. Consequently, new species with only minor phenotypic adaptations occur. This phase is characterized by a more gradual pattern of evolution proceeding in an orthogenetic direction. An example for the limited phenotypic change is the transition from the Przewalski to the domestic horse (Yang et al. 2003). **c** Typolysis: at the end of the line, once all acrocentric chromosomes fused and became metacentric chromosomes, the lineage either undergoes a massive splitting of metacentric chromosomes and starts again or dies from chromosomal instability. Eroded telomeres on many chromosomes lead to a burst of transposon activity. According to the fossil record, pseudovariability and degenerative diseases characterize species phenotypes, shortly before their complete disappearance. During the lengthy typostatic phase, eventually new evolutionary cycles branch off due to the combination of chromosomal aberrations and significant transposon-mediated genomic repatterning

At the end of a chromosomal centric-fusion-sequence in a lineage, I agree with N.B. Todd that simultaneous fissions must occur (Todd 1970), resetting the karyotype to a mostly acrocentric state (Fig. 5). In such cases of fissioning, a mechanism is required that rebuilds and elongates telomeres on the newly formed ends of acrocentric chromosomes. Since the first mammals in ancient history looked very similar to modern shrews, it is startling that the longest telomeres have been reported in the shrew *Sorex granarius*. All telomeres near the centromeres of the acrocentric chromosomes of this shrew species are up to 300 kb long (Zhdanova et al. 2007), which is 30× times longer than human telomeres. Yet, telomeres on the opposite ends of the same chromosomes of this species are in the range of the all-metacentric shrew species *Sorex araneus/novosibirsk*, whose telomeres are all relatively short (Zhdanova et al. 2005, 2007). Mitochondrial DNA of these two species indicates a very close relationship (Taberlet et al. 1994), in contrast to the massively reorganized karyotypes, which is all-metacentric in *S. araneus/novosibirsk* and largely acrocentric (except for two metacentrics) in *S. granarius*. Accordingly, the huge telomeres on the proximal ends of *S. granarius* chromosomes favor a scenario where *S. granarius* is the outcome of a massive fission event in a population of *S. araneus* (Zhdanova et al. 2005) followed by a process of enormous telomere elongation. Based on the finding that the very long telomeres of *S. granarius* also contain interspersed sequences of ribosomal DNA (Zhdanova et al. 2007), it is tempting to speculate on even multiple chronological triggers of transposon-mediated repatterning during the replicative erosion of one telomere. *Sorex araneus* exhibits one of the most outstanding chromosomal polymorphisms found in mammals and a clear trend toward a segregatic distortion in favor of metacentrics during male meiosis has been found (Wyttenbach et al. 1998). This kind of meiotic drive is most likely a consequence of the negative effect of eroded telomeres of acrocentrics on sperm fitness. For this reason, male germ cells with metacentric fusion products may be selected.

**Fig. 6:**
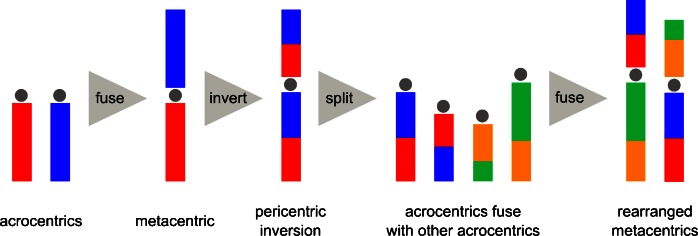
Centric fusion–fission cycle in combination with pericentric inversions as the theoretical model for rearranged karyotypes as seen in gibbons compared to humans. In contrast to current mainstream models, no translocations between different chromosomes are required, which bear a higher risk for unbalanced offspring

Based on his extensive chromosomal studies, White speculated on the continued reappearance of a particular karyotype within lineages, caused by selection for some kind of equilibrium (White 1973, p. 453). However, I want to emphasize that after the splitting of metacentrics, some acrocentrics may be structurally different compared to their ancestral counterparts at the beginning of the cycle, because of pericentric inversions that occurred in some of the ancient metacentric chromosomes (Fig. 6). If these rearranged acrocentrics fuse again during later evolution of a lineage with other rearranged acrocentrics, the resulting karyotype might look quite similar to that of the gibbons in relation to humans (Fig. 2). Consequently, centric fusion, pericentric inversion and centric fission might be the dominant forms of chromosomal change in mammalian evolution.

## What defines the phenotype of a new species — gene mutation or gross chromosomal aberration or transposon-mediated repatterning of the genome?

It can be frequently observed that if gene sequencing data do not comply with the reality of two species, because the genetic variation between individuals of one species is sometimes equal to or even greater than between the two species (De Grouchy 1987; Sharp et al. 2006), incomplete lineage sorting and ongoing interspecies hybridizations are proposed. Besides its major blow to the concept of genotype–phenotype correlation that supposedly determines a species, this defense strategy is easy to disprove based on the overwhelming knowledge of differing chromosome complements causing reproductive barriers. Let us start with the gibbons and show why interspecies hybridizations generally result in dead ends. Kim et al. (2011), in a large study on nuclear sequence variation within and between gibbon species, found that *Nomascus leucogenys* and *Nomascus gabriellae* are genetically so similar that the authors proposed an ongoing gene flow. However, the karyotypes of these two species can be distinguished by at least two gross chromosomal rearrangements, a pericentric inversion and a translocation between two chromosomes (Couturier and Lernould 1991). Every doctor who is specialized in genetic counseling knows that if one of the parents were homozygous for a large translocation and an inversion, family planning would become a serious challenge (De Braekeleer and Dao 1990). One hundred percent of the offspring would be heterozygotic carriers of both rearrangements and consequently would have a significantly reduced fertility (Walsh 1982). Therefore, the genetic sequence of one species can never effectively spread to the population of another species, if cross-chromosomal differences are present (which is usually the case between species (White 1978)). The results of a study on human–chimpanzee genetic divergence tell a similar story. Divergence time varies by about 4 million years along the DNA sequences, with the X chromosome being extraordinarily similar in sequence. Again, the authors of a *Nature* article propose sporadic rehybridizations after the initial split (Patterson et al. 2006). Unfortunately, the authors of this *Nature* piece have overlooked an important detail — How probable is the hybridization between lineages, whose living representatives differ in one fusion of two chromosomes and nine large pericentric inversions (Ijdo et al. 1991; Szamalek et al. 2006)? Even if not all of these gross chromosomal differences existed from the start, based on the ABC of genetics, this reproductive barrier is solid and hybridizations between apes and humans with complete introgressions are highly unlikely. In agreement with the aforementioned, the rate of genetic divergence between humans and chimpanzees was not accelerated in rearranged parts of chromosomes and therefore the authors concluded that hybridization between incipient species of these two lineages did not occur (Zhang et al. 2004). Besides, any human or ape usually knows what its own kind looks like and consequently limits its mating attempts to members of its own kind. In my view, the currently favored admixture and interbreeding concepts, which seem to be based on “mating with everything that moves”, can never be a widespread mechanism of evolution.

Have you ever heard of *Trichomonas vaginalis*? You should have. According to molecular genetics, this is the most advanced eukaryote. Whereas humans make do with only 20,000 genes, this single-celled parasite living in the urogenital tract of humans seems to have 60,000 protein coding genes, according to a draft genome sequence (26,000 genes had similarities with known proteins or expressed sequence tags in other eukaryotes; however, the function of the majority of the other predicted genes is unknown) (Carlton et al. 2007). Based on the recent discovery of many of these phenotype–genotype paradoxes, the logical deduction that protein-coding genes do not define the phenotype of a species is inevitable. The disappointment about the frequent discovery of nuclear sequences not reflecting the differences of species phenotypes lead some scientists to propose differential gene expression as the source of phenotypic change. However, the rather depressing results of a gene expression study between tissues of human and chimpanzee with the testis being the organ with the greatest difference in gene expression patterns and with the brain displaying the lowest, further dampened the euphoria somewhat (Kehrer-Sawatzki and Cooper 2007).

Based on the aforementioned, we have to conclude that gene mutation and gene expression patterns do not directly determine the phenotype of a species. What about gross chromosomal changes? An important argument in favor of the chromosomal theory of speciation is that all members of a species are characterized by identical autosomal chromosomes each in a homozygous state, with the exceptions of polymorphisms and sporadic spontaneous aberrations (a few per thousands) (De Grouchy 1987). However, as Max King put it: “There seems to be little doubt that the only valid role that chromosomal change can have in speciation is that of a reproductive isolating mechanism (…). There is no evidence to suggest that chromosome change is directly responsible for morphological change in speciation” (King 1993, p. 272). If one considers the huge number of chromosomal races of house mice with only modest phenotypic differences (Corti and Rohlf 2001) and the huge phenotypic variation of the domestic dog without even one chromosomal change, Max King clearly is right. However, molecular genetic variation between dog breeds is in the range of that of humans (Shearin and Ostrander 2010) and cannot explain the diversity of dog phenotypes either.

As James A. Shapiro emphasized, the triplet code for amino acids in proteins is not the only genetic code, there are others for packaging, replication, distribution and evolution. Based on Shapiro’s theoretical work, it is tempting to suggest that there is some kind of information stored in the repetitive transposon-containing parts of the genome of higher organisms. There must be some sort of code, similar to the triplet code. Species-specific transposons have been shown to rewire the transcriptional network in mouse and men (Kunarso et al. 2010) and some have suggested the term “transposon code” (Testori et al. 2012). In eutherian mammals, the promoter of prolactin expression in the endometrium of the uterus has evolved at least three times, in each case by the utilization of a different kind of retrotransposon (Emera and Wagner 2012). Yet, endometrial prolactin is essential for a variety of functions during pregnancy and it is therefore highly unlikely that its controlled expression through a transposon-derived promoter occurred several times just by accident during evolution. Since early eutherian evolution, about half of the ancestral functional bases have been turned over, and half the current functional bases have been gained (primarily transposon-derived), whereas the protein-coding complement evolved relatively slowly (Ponting et al. 2011). Therefore, I am convinced that focusing on the transposon-derived repetitive sequences may one day pave the way to the understanding of the body plan of higher organisms and the material basis of phenotypic evolution. In analogy to the binary code of modern computers, the elements of the repetitive sequence might represent the bits of stored information.

So what defines the phenotype of a new species? The Danish embryologist Søren Løvtrup said: “Evolution is not a question of making new materials, but rather of using old materials for new purposes” (Lovtrup 1987, p. 376). Similarly, Shapiro writes: “Evolution appears to proceed by the utilization of basic biochemical routines in different combinations in different organisms. With few exceptions, the structural proteins of all mammals are probably interchangeable; what makes a mouse different from an elephant is when and how those molecules are synthesized and assembled during development” (Shapiro 1992). “Repetitive DNA is far more taxonomically specific than protein-coding DNA and serves as the most reliable indicator of identity for a species or even, as used in forensic analysis, for identifying an individual” (Shapiro 2002). I agree with Shapiro on the significance of transposon-mediated genomic repatterning, which may instantaneously reset the body plan in the germ cells of higher organisms. In contrast to Shapiro’s model, I suggest transgenerational telomere erosion to be the trigger for that saltational genotypic and phenotypic change in several individuals at once.

## Summarizing the theory and applying it to Schindewolf’s model of typostrophism

The variety of chromosomal rearrangements, which occurred during organic evolution, is enormous. However, nonrandom sequences of chromosomal change are frequently seen. One of them is the centric fusion–fission cycle of acrocentric chromosomes in combination with pericentric inversions in metacentric chromosomes. Let us choose this common type of chromosomal evolution to check if our line of thoughts is compatible with the reality of the fossil record. My theoretical model predicts that ever-shortening telomeres between generations of a species lead to uncapped chromosome ends near the centromeres of acrocentric chromosomes, in a nonrandom manner (Stindl 2004a). Two acrocentrics fuse and form a metacentric chromosome in germ cells of many individuals of a species possibly within a single generation. Their descendants represent a new chromosomal race or species with two newly formed homologous metacentric chromosomes and a lowered chromosome number by two. In addition to chromosomal fusions, telomere erosion triggers the activation of transposable elements, which result in numerous genomic rearrangements. Transposons can lead to gross chromosomal changes, like pericentric inversions and translocations, which cause reproductive barriers (as chromosomal fusions and fissions do), but transposable elements also produce numerous submicroscopic rearrangements, like deletions, duplications and inversions, which are thought to be responsible for the phenotypic change. Thousands of minute structural genomic differences that separate species, like chimp and human, have been reported (Cheng et al. 2005; Feuk et al. 2005; Lee et al. 2008), which I propose to be transposon-mediated, triggered by eroded telomeres. In some cases of speciation, telomere erosion might trigger submicroscopic transposon-mediated genomic rearrangements even without gross chromosomal changes. However, these new species with unaltered karyotypes might lack a robust reproductive barrier to the old species.

Those who question that such massive cutting of the genome is possible in the germline of an individual, I want to remind of the ubiquitous mechanism of meiosis. Hundreds of programmed double-strand breaks occur in homologous chromosomes during the production of germ cells in higher organisms to generate the genetic variation between individuals (Kauppi et al. 2013). I postulate an analogous mechanism for the repatterning of the genome that defines the phenotype of a new species.

Chromosomal fissions or other structural aberrations, which result in newly created chromosomal ends, require a mechanism, which rebuilds and elongates telomeres. Several scenarios for the creation and/or elongation of telomeres are possible:

- According to the recent literature, the enzyme telomerase seems incapable of elongating telomeres to the extent required for the new model. The main function of telomerase has been shown to be one of a stabilizer of telomere length in germ, embryonic and cancer cells (Stindl 2008). In my view, the most promising candidate is a mechanism called alternative lengthening of telomeres (ALT), which has been found in a subset of tumors and tumor-derived cell lines to significantly extend telomeres by >50 kb. ALT activity has only been seen in abnormal situations and in the presence of dysfunctional telomeres and may represent the improper up-regulation of a normal cellular pathway (Cesare and Reddel 2008).
- Telomere creation and elongation could be carried out in a similar (yet unknown) manner as the periodic amplification of satellite DNA during evolution (theory of genome resetting (Gillespie et al. 1982)). Very large blocks of interstitial telomeric sequences, which have been observed on certain metacentric chromosomes in some species (Go et al. 2000) might, therefore, not be remnants of a chromosomal fusion event, but preparations for an evolutionary split event.
- Telomeres could be newly built and elongated by transposon-mediated mechanisms. An extreme example of the transposon–telomere connection is *Drosophila*, with telomeres consisting entirely of retrotransposons instead of the repetitive telomeric DNA sequence (Levis et al. 1993).
- The population bottleneck in the parent generation of the old species (due to telomere-driven morbidity and mortality) might increase the level of inbreeding in the first generation of the new species. Inbreeding has been thought to be responsible for a 10× times lengthening of telomeres in lab mice compared to their wild counterparts (Manning et al. 2002; Stindl 2004a) and could be an evolutionary mechanism to rebuild telomere length in a new species (Stindl 2004a).

Let us now have a look at how the proposed biological mechanisms fit into the theory of typostrophism (Fig. 5).

Schindewolf’s typolytic phase: Eventually, in species with all-metacentric karyotypes (and many critically short telomeres), a transposon-mediated fissioning of metacentric chromosomes occurs. Due to numerous critically short telomeres, transposon activity is high and consequently the genome is in an unstable state. Independent of the occurrence of fission events, if telomeres cannot be stabilized, the lineage becomes extinct.

Schindewolf’s typogenetic phase: After the splitting of metacentric chromosomes in the typolytic phase, telomeres are rebuilt and lengthened at the beginning of the typogenetic phase, by mechanisms listed above. The chromosomal complement is reorganized and stabilized in the surviving lineage. The high levels of transposon activity are responsible for massive repatternings of the genome and consequently a speciation burst. The abundance of acrocentric chromosomes in combination with transgenerational telomere erosion is the basis for a new typostrophic sequence.

Schindewolf’s typostatic phase: The trend towards metacentricity starts again with the stepwise fusion of specific acrocentric chromosomes leading to new chromosomal races (no significant phenotypic change) or new species (telomere-triggered transposon-mediated repatterning). This phase is characterized by a more gradual pattern of evolution, but still not in the sense of neo-Darwinism, but in an orthogenetic way. During the lengthy typostatic phase, new evolutionary cycles can branch off due to the combination of chromosomal aberrations and significant transposon-mediated genomic repatterning. The Italian zoologist Rosa was the first to observe that the amount of morphological novelty steadily declines in a lineage (=Law of progressively reduced variation (Luzzatto et al. 2000)). Accordingly, I propose that at the beginning of the typostatic phase many different combinations of chromosomal fusions are possible (followed by transposon-mediated repatternings), whereas at the end there are only a few acrocentrics left (Fig. 5). During the typostatic phase, telomere elongation is not essential, since the unstable telomeres disappear after each chromosomal fusion.

To explain Cope’s law of unspecialized descent, namely, that new types seem to derive from the most primitive representatives of the ancestral type (Schindewolf 1993, pp. 256–257), I present an analogy to the digital world. Resetting the genome during evolution might be analogous to a reinstallation of Microsoft Windows on an old PC, where you lose all personal settings and the operating system restarts with the initial standard setup provided by the manufacturer. Accordingly, I propose that new types directly develop from specialized types, but because of complete genomic resetting during the typolytic and typogenetic phases the phenotype of the new type can be unspecialized and primitive.

Both theories — the species clock hypothesis and the telomeric sync model of speciation — emphasize the central role of an intergenerational telomere clock in triggering chromosome fusions and transposon-mediated genomic rearrangements, which either lead to speciation or extinction (Table 1). It is important to note that in addition to the widespread centric fusion–fission cycle, all other observed patterns of chromosomal evolution can theoretically be carried out by these cell biological mechanisms. For example, the male Indian muntjac deer has seven huge chromosomes (six in diploid females), which are mostly the result of numerous tandem fusions of chromosomes of an ancient karyotype similar to the closely related Chinese muntjac with 46 chromosomes (McClintock 1984). As in other cases of chromosomal fusion, supernumerary centromeres are deactivated in the chromosomes of the Indian muntjac, because di- or multicentric chromosomes would be unstable during mitosis.

**Table 1:** Problem-solving capacity of the proposed concept

Problem	Solution
99.9 % of species died out (Raup 1991)	Internal cause due to transgenerational erosion of telomeres (=species clock)
Saltatory pattern of speciation in the fossil record	Telomere erosion leads to chromosomal fusions and triggers a transposon-mediated repatterning of the genome (=telomeric sync model of speciation)
Significant phenotypic change between species despite only insignificant changes in protein-coding sequence	Eroded telomeres trigger a transposon-mediated repatterning of the genome that rebuilds the body plan
Underdominance of a spontaneously occurring chromosomal aberration in a heterozygotic individual prevents its spread in a population	Observed species-specific telomere profile leads to fusion of identical acrocentric chromosomes and identical transposon-mediated repatterning in many individuals at once
Observed meiotic drive of de novo Robertsonian translocations in males	Consequence of eroded telomeres on acrocentrics and the selection for sperm cells with newly rearranged metacentrics with stable telomeres
Obvious lack of isolation genes (Wu and Ting 2004)	Identical chromosomal fusions and transposon-mediated chromosomal rearrangements (pericentric inversions, translocations and others) occur simultaneously in many individuals and are effective reproductive barriers to the old species
Neanderthal genes found in modern humans, but not vice versa. Yet, the standard model of hybridizations between archaic and modern humans would predict a bidirectional gene flow	Alternatively, the multiregional model with local transformations of archaic into modern humans can better explain why no modern genes are found in Neanderthal DNA

## The last heresy — intrinsic causes of species extinction

Of all the species that have existed at one time or another on earth, only about 1 in 1,000 is still alive; hence 99.9 % of species died out (Raup 1991, pp. 3–4). Clearly, according to Darwin’s theory, the causes of extinction must usually lie outside of the organism, because prospering species have been adapting for many thousands of years and must be very fit. As a consequence, all kinds of threats to species survival have been proposed — mostly humans, climate change, asteroids and limited food resources. Yet, in the Pleistocene, the average American did not have access to automatic fire weapons, craters of asteroid impacts have not been found and the climate (if ever) changed in a rather smooth way, which leaves us with the idea of limited food supply. Consequently, some authors have suggested that limited prey resources forced saber-toothed cats and American lions to utilize more of the remaining carcasses leading to a greater incidence of tooth breakage. The story goes that times were difficult and therefore both predominant carnivores became extinct 12,000 years ago. The Rancho La Brea tar seep deposits in California, representing the past 50,000 years, provide an abundance of remarkably well-preserved specimens. Based on the fossil record, rates of tooth breakage increased in both examined species until extinction. Especially in the American lions, shortly before extinction, a stunning 36 % were affected by this dental handicap. However, Desantis et al. (2012) clearly showed that the diet did not change, and extensive bone crushing cannot be the cause of dental degradation in these large carnivores. Schindewolf preferred an alternative explanation in his 1950 book and cited the Austrian paleontologist Othenio Abel, who described the very abundant evidence of Ice Age cave bears, which, shortly before becoming extinct, exhibited extremely wide variability and all kinds of manifestations of degeneration, including severe bone and tooth disease and injuries — even young animals were affected by these conditions (Schindewolf 1993 p. 322). Abel was convinced that degeneration of a species was a consequence of optimum existence (Abel 1980). Yet, Schindewolf notes: “The symptoms of the cave bears are strikingly similar to the disease-altered bones that Hansen described from Norman graves in Greenland” (Schindewolf 1993 p. 322). In contrast to the cave bears extinction, in Greenland living conditions seemed to be worsening during that time. Consequently, Schindewolf states: “We arrive at the same conclusion, that the actual causes of degeneration and extinction lie deeper and manifest themselves earlier than any environmental influences whatsoever” (Schindewolf 1993, p. 323). “Thus, the reasons for extinction or continued existence are essentially internal — they lie within the lineages themselves. As P. Jensen, C. Zimmer, Karl Beurlen and other authors believe, the reasons may perhaps be sought in an aging of the germ substance, a gradual loss of function in the sex glands resulting in reduced fertility” (Schindewolf 1993, p. 322). According to Otto H. Schindewolf, geological catastrophes would be only the last hit, putting an end to a process that had been underway for ages for internal reasons. “It is the same as when the wind finally topples an old and rotten tree” (Schindewolf 1993, p. 319).

Cope’s Rule, the observed tendency for organisms in a lineage to increase in body size over time, is still poorly understood in terms of selection for fitness, especially in the many cases of gigantism. Alternatively, Schindewolf claimed that orthogenesis, the primary trend of evolution, is to blame. First, it yields a normal, beneficial size increase and later inevitably exceeds it and leads to a serious disadvantage and even to extinction of a species (Schindewolf 1993, p. 309). Since a larger body size requires more cell doublings, especially during lifelong regeneration of somatic tissues (Stindl 2004b), it is easy to imagine how increasing height in a lineage can have a negative effect on telomere reserve.

Conflicting literature data on the mean telomere length of somatic tissues and its consequences for aging and age-associated diseases have been reported over the years. In my view, this has two main reasons: Almost all researchers investigate the telomere length of blood samples, despite the fact that mammalian red blood cells lack a cell nucleus and only telomeres of the small fraction of white blood cells can be measured. These immune cells show complex patterns of migration and replenishment, which are influenced by various factors (e.g., stress) and might, therefore, not provide a reliable picture of the telomere reserve of an individual. The other shortcoming is the widespread ignorance of the mechanisms of somatic tissue regeneration by adult stem cells (Stindl 2008). Consequently, I suggest that telomere length and the available number of adult tissue stem cells in a given species might be the determining factors of lifespan, regeneration capacity of tissues and aging.

An anecdotal case of an endangered pack of wolves with unusual signs of aging and degeneration on Isle Royale in Michigan was recently reported in *Science* (Mlot 2013). The population was established six decades ago and remained stable until the 1980s when a viral disease reduced their numbers to a mere dozen. In 1997, a large male wolf from Ontario crossed the ice bridge. This wolf became whiter as he aged, something not seen before in Isle Royale wolves. He sired 34 offspring and genetically took over the population. Over the last years, physical abnormalities have increased to abnormal levels. In 2009, the majority of the wolves had some kind of spinal deformities. Another mystery is the occurrence of several wolves with one opaque eye, not seen before. Finally, in summer 2012, no pubs were born and the remaining population of four female and four male wolves now faces extinction. Some researchers at Isle Royale blame inbreeding for the signs of degeneration (Räikkönen et al. 2009), although an opaque eye is usually a result of aging, not inbreeding. Telomere length measurements might bring new aspects into the discussion.

A high percentage of ancient Egyptians were considerably crippled, by changes in the vertebral column and by lesions of the peripheral articulations (Ruffer 1919; Moodie 1923). Similarly, 38 % of ancient Egyptians and 25 % of ancient Peruvians with a mean age at death of around 40 years showed signs of atherosclerosis (Thompson et al. 2013). Unfortunately, an accurate chronological survey of cases of degeneration and atherosclerosis is not possible based on the findings of these studies. Yet, it was shown that the health of pre-Columbian populations significantly deteriorated long before Columbus arrived and climatic distinctions were completely irrelevant. Surprisingly, hunter–gatherers, who lived several millennia ago, were the healthiest Native Americans in stark contrast to the later people, who lived in times of agriculture, government and urbanization. About 90 % of the aboriginals may have died within the following two centuries after the arrival of the Spanish; however, the authors found the long-term trend towards a poor health status of aboriginal populations to be the causal factor of the speed and ease of the conquest (Steckel and Rose 2002). Clearly, a thorough reexamination of all signs of degeneration in troubled species or populations is needed, to put the new theoretical model of an intrinsic extinction mechanism on solid scientific grounds.

In accordance with the aging of the germ substance idea cited by Schindewolf, the telomeric sync model of speciation is based on transgenerational telomere erosion, which can lead to decreased fertility (Baird et al. 2006) and an increase of age-associated (Sharpless and DePinho 2007) and all sorts of degenerative diseases (Chang et al. 2004) at the end of a species lifespan. Age-associated diseases, like cancer, cardiovascular disease, immunosenescence and dementia, and degenerative diseases of teeth, bones and joints, are proposed to culminate even in middle-aged individuals and fertility decreases. Critically short telomeres in somatic tissues and in germ cells of individuals have been shown to be capable of causing all these kind of health issues. During such a transformation phase, the species either transforms into a new species, or stabilizes its telomeres, or becomes extinct. However, since the phenotypic change, triggered by short telomeres and mediated by transposons, can be enormous, the close relationship of two species might be invisible in the fossil record. Consequently, the extinction of some species might be an artifact of the fossil record caused by the gradualistic genetic model of evolutionary theory. It is my conviction that some extinctions, like the ones of the Neanderthals, will one day turn out to be transformations.

## Hominin evolution: extinction and complete replacement of archaic humans worldwide … really?

Some years ago, the evolution of hominins was every neo-Darwinist’s darling. It was all about an African progressing series of archaic hominins resulting in the superbright *Homo sapiens* spreading out of Africa and replacing all other dumb relatives worldwide. Nowadays, according to Kimbel the story reads differently: “The evolutionary events that led to the origin of the *Homo* lineage are an enduring puzzle in palaeoanthropology.” (Kimbel 2013) What happened? Well, it turned out that instead of a gradual phenotypic change towards perfection, nature seems to have played around with different combinations of “archaic” and “modern” body parts that make no sense under the light of genetic gradualism. A series of reports published this year in *Science* focused on fossilized skeletons of *Australopithecus* (Kimbel 2013). One sample of *Australopithecus afarensis* has an upper thorax more similar to modern humans, although it is 1.6 million years older than *Australopithecus sediba*, which has an ape-like pectoral girdle (Kimbel 2013). Furthermore, the fossilized skeletons of *A. sediba* had a surprisingly ape-like calcaneus, in contrast to its *Homo*-like mandibles. To further complicate matters, although *Australopithecus* is usually characterized by six lumbar and four sacral vertebrae, in *A. sediba* the modern human pattern is seen, which is five lumbar and five sacral vertebrae (Kimbel 2013).

If the phenotypic confusion in the hominin lineage still leaves some unconvinced, let us turn to comparative sequencing data. Based on the fact that mitochondrial DNA of all Neanderthal specimens falls outside the variation of present-day humans, interbreeding between archaic and modern humans was a no-go for many years (Ward and Stringer 1997). However, the draft sequence of the Neanderthal genome clearly confirmed archaic genes in our genome and the authors suggested a unidirectional gene flow from Neanderthals into the non-African ancestors of present-day humans before the Eurasian split (Green et al. 2010). In the same year, the sequencing of the DNA extracted from a finger bone led to the birth of a new archaic cousin in southern Siberia, the Denisovan (Reich et al. 2010). Again, it was found that the Denisovan man, similar to the Neanderthal, contributed 4–6 % of its genetic sequence to modern humans, although to Melanesians only (Reich et al. 2010). A 100-year-old lock of hair from an Aboriginal man in southern Western Australia revealed similar admixture rates with archaic humans (Rasmussen et al. 2011). And so, it was concluded that *Homo sapiens* interbred with now-extinct forms of humans all over the world (Gibbons 2011). According to a 2011 *Science* study, more than half the HLA alleles of modern Eurasians must have introgressed due to multiple and widespread admixtures with archaic humans. The authors suggested that the surprisingly high numbers were the consequence of some sort of selection (Abi-Rached et al. 2011).

Yet, if one considers that the reproductive barriers of different chromosome complements effectively prevent the vertical spread of foreign genes in a population, it remains an eternal mystery how the proposed sexual activities of our immediate ancestors with all kinds of archaic hominins could result in significant numbers of fertile offspring. Of course, we do not know the karyotype of Neanderthals or Denisovans, but differing chromosome complements are the hallmarks of closely related species (White 1978; Cho et al. 2013) and extinct hominins were successful and independent species for many thousands of years. Besides the surprising sequencing data, the paleoanthropologists have always pointed to certain bone and dental features of Neanderthals that apparently survived in all modern Europeans (Trinkaus 2007). Already in 1943, Franz Weidenreich, one of the early proponents of the multiregional model, wrote: “Two years ago I published an article (…) dealing with the obvious incongruities of the morphological and chronological sequences of the various evolutionary stages of Man as they appear on the basis of steadily increasing discoveries of recent years. At the very appearance of true hominids there must have already existed several different branches, morphologically well distinguishable from one another, which all proceeded in the same general direction with mankind of today as their goal” (Weidenreich 1943).

The telomeric sync model of speciation predicts successive series of defined chromosome rearrangements and genomic repatternings in all individuals of a species within similar time intervals, worldwide. Accordingly, the remains of archaic genes and phenotypic traits found in modern humans, typical for local archaic hominins, might be a consequence of directly developing from these local ancestors through a defined genomic repatterning. The telomeric clock that triggers programmed rearrangements and transposon-mediated repatternings in combination with worldwide gene flow within a species might be responsible for the proposed 99.9 % sequence identity in human populations around the world, despite the separate development of local lineages for many thousands of years. Clearly, the unexpected finding of a unidirectional gene flow from Neanderthals into modern humans only, but not in the other direction (Green et al. 2010; Wills 2011), inevitably supports the multiregional concept of local archaic humans directly transforming into modern humans. In other words, there were no other local and healthy archaic humans left to interbreed with, once the new generation of modern humans evolved from them. It is an indisputable fact that the observed one-way genetic exchange from archaic to modern humans shakes the foundations of the currently favored admixture and interbreeding model, which is thought to result in some sort of bidirectional gene flow. For all these years, modern humans have been regarded as being superior to their archaic counterparts, and now, even if the standard population genetic model predicts a bidirectional gene flow with a dominating modern-to-archaic direction, the neo-Darwinists suddenly discover the exclusive superiority of archaic genes from a dying human lineage. The Danish embryologist Søren Løvtrup once commented: “And today the modern synthesis (…) is not a theory, but a range of opinions which, each in its own way, tries to overcome the difficulties presented by the world of facts” (Lovtrup 1987, p. 144).

## The transformation or bifurcation phase as exemplified in Finnish blue foxes and humans

In a Finnish farm, several hundred blue foxes, parents and offspring, were analyzed over 4 years. About half of them had a Robertsonian translocation in a heterozygous form (2*n* = 49), whereas a quarter were homozygous carriers (2*n* = 48) and a quarter had the original karyotype with two acrocentrics (2*n* = 50). As expected and predicted by genetics, litter size tended to be smaller in mating groups of chromosomal heterozygotes in this study (Makinen and Lohi 1987), in contrast to a previous report (Moller et al. 1985) but in line with an older study (Christensen and Petersen 1982). Surprisingly and contrary to the predictions, animals with the Robertsonian translocation in a homozygous form (2*n* = 48) increased over the 4-year span (Makinen and Lohi 1987). Accordingly, it was observed that matings of two heterozygotes seemed to favor the 2*n* = 48 offspring production (Makinen and Lohi 1987) and the spread of a new chromosomal race. It was therefore shown that the blue fox displayed an evolutionary tendency towards a lower chromosome number and that the Robertsonian translocation in its homozygous form had a positive effect on fertility. If, after 30 years, this farm still exists and breeders have not intervened based on karyotypes, a re-examination of the descendants of these animals would be an interesting project.

In humans too, Robertsonian translocation (ROB) is the most common recurring chromosomal rearrangement. *De novo* formation of fusions between chromosome 13 and 14, rob(13q14q), accounts for the largest proportion of ROBs (Page and Shaffer 1997). Jacobs states: “The reason for the high mutation rate of human Robertsonian translocations in general, and for the 13/14 translocation in particular, is obscure” (Jacobs 1981). Several scenarios have been put forward to explain this phenomenon. Bandyopadhyay and colleagues proposed illegitimate recombination between paralogous satellite III DNA on acrocentric chromosomes. They classified ROBs into two groups: Class I, mainly rob(13q14q) and rarely rob(14q21q), account for 85 % of ROBs, and class II includes all other sporadic ROBs. Breakpoints of the common class I ROBs are almost always in the same region, whereas sporadic class II ROBs are characterized by varying breakpoints. Regarding these class II ROBs, the authors write: “The variable breakpoint could result from breakage and exchange in repetitive DNA, such as satellite III DNA sequences, that are common to all acrocentric short arms and the pericentromeric regions of these chromosomes” (Bandyopadhyay et al. 2002). Since a sporadic breakpoint within repetitive DNA would always vary, and this is not seen in the majority of common ROBs (Bandyopadhyay et al. 2002), I conclude that illegitimate recombination between paralogous satellite III DNA cannot be the source of common human ROBs. Another mechanism, which has been put forward, is based on the fact that acrocentric chromosomes come physically near to form the nucleolus, because of rDNA genes. However, human rDNA genes are located on the short arms of all acrocentric chromosomes (13, 14, 15, 21, and 22; Henderson et al. 1972) and cannot explain why just two combinations, which are rob(13q14q) and rob(14q21q), are constituting 85 % of all ROBs, why the breakpoints are almost always in the same region and why 95 % of de novo cases originate during maternal meiosis (Page and Shaffer 1997; Bandyopadhyay et al. 2002).

Rescue comes from the observation of nonrandom telomere patterns in humans (Graakjaer et al. 2006) and the indirect evidence of telomere erosion in the female germline (see above). In a small study on 20 aged individuals, the telomere on the proximal end of chromosome 13 has been found to be the shortest and on the p-arms of chromosome 14, 15 and 21 one of the shortest (Graakjaer et al. 2006). Except for the short telomere on 15p, the telomere data fit the observed pattern of fusion products.

Evidence for a prezygotic selection for ROBs in male humans has been described, similar to the meiotic drive of ROBs in the common shrew we discussed earlier (Hamerton 1968). Again, I propose the negative effect of short telomeres on fitness to be the underlying cause. Sperm cells containing a rearranged metacentric chromosome instead of two acrocentrics with eroded and unstable telomeres may simply be preferred. Based on the suggested telomere erosion in the human species and the nonrandom telomere profile, we would expect to see the appearance of a new chromosomal race, with 44 chromosomes and two rob(13q14q). Is there any evidence for such a transformation or bifurcation phase? During a cytogenetic study of an aged population, a heterozygous carrier of a rob(13q14q) was found. He was 90 years old and in good general health. The authors mentioned that he looked younger than his chronological age and that all his close relatives survived beyond the age of 80 (Anday et al. 1974). In 1984, Martinez-Castro and colleagues were the first to report on a Spanish family with heterozygous and homozygous carriers for a rob(13q14q) without any impairments of phenotype. They also observed an excess of homozygous carriers among the progeny of heterozygotes, in accordance with prezygotic selection for ROBs (Martinez-Castro et al. 1984). The stunning discovery was confirmed by a Finnish study based on three families with a female again being homozygous for rob(13q14q) and a karyotype of 44 chromosomes. The authors described the good health and normal phenotype of these individuals and speculated that rob(13q14q) might be the next step in the chromosomal evolution of man (Eklund et al. 1988). Clearly, a re-examination of these families is highly recommended. Furthermore, I suggest to undertake a cytogenetic survey of the 100 or so isolated aboriginal human populations worldwide, to measure telomere length and to search for alternative chromosomal races.

## Conclusions

In this paper, I present an alternative to Darwin’s gradualistic theory and provide a biological framework for the old European concept of saltatory evolution, summarized best in Otto H. Schindewolf’s book, *Basic Questions in Paleontology* (Schindewolf 1993). The high quality of the fossil record in sediments of ancient oceans guarantee that Schindewolf’s extensive studies of corals and cephalopods are superior to the currently dominant genetic models of modern laboratory-based scientists. As a consequence, my telomeric sync model of speciation mainly builds on Schindewolf’s typostrophic theory. In short, I propose that transgenerational telomere erosion leads to identical chromosome fusions and triggers a transposon-mediated genomic repatterning in many individuals at once. The phenotypic outcome of the telomere-triggered and transposon-mediated repatterning is the saltatory appearance of nonadaptive characters in new species, which is in perfect agreement with the fossil record (Table 1). The species clock based on transgenerational telomere erosion gives species a sense of time and is therefore the material basis of aging at the species level. According to the telomeric sync model of speciation, speciation events can be triggered suddenly and simultaneously, eventually synchronizing the transformation of a whole interconnected biotope of many plant and animal species within a relatively short time frame.

In addition to the studies and experiments I have already put forward to test the proposed model, the currently observed immunodeficiency of honeybees displays several signs of a telomere-driven species crisis (Stindl and Stindl 2010). A study of telomere length and chromosomal races in affected honeybee populations is therefore highly recommended. Similarly, the white-nose syndrome of North American bats should be reinvestigated in the light of telomere-driven immunosenescence (Buchen 2010). However, I have to point out that measuring mean telomere length is not sufficient because a single critically short telomere determines the viability of a cell (Hemann et al. 2001), possibly the life expectancy of an individual and according to the new evolutionary model, the duration of a species.

Regarding the widespread misconception of the superiority of experiments over theoretical models in biological research, I have to conclude with one of Richard Goldschmidt’s very thought-provoking remarks: “A good observation may lead to results which a meaningless experiment cannot achieve, and a good idea or analysis may accomplish with one stride what a thousand experiments cannot do. This truism, obvious as it is in the history of all sciences, is frequently forgotten in this era of overestimation of new techniques, which are tools of progress only when in the hands of constructive thinkers” (Goldschmidt 1982, p. 184).
